# Single cell transcriptome profiling reveals cutaneous immune microenvironment remodeling by photodynamic therapy in photoaged skin

**DOI:** 10.3389/fimmu.2023.1183709

**Published:** 2023-06-19

**Authors:** Yu Yan, Guorong Yan, Zhi Cao, Bo Wang, Qingyu Zeng, Lei Shi, Qihang Chang, Chengqian Chen, Linglin Zhang, Caihe Liao, Shengkai Jin, Xiaofei Sun, Guolong Zhang, Peiru Wang, Xiuli Wang

**Affiliations:** ^1^ Institute of Photomedicine, Shanghai Skin Disease Hospital, Tongji University School of Medicine, Shanghai, China; ^2^ Department of Dermatology, University of Michigan, Ann Arbor, MI, United States; ^3^ Department of Dermatology, Huadong Hospital, Fudan University, Shanghai, China

**Keywords:** ALA-PDT, single cell RNA sequencing (scRNA-seq), photoaging, immune microenvironment, immunosenescence

## Abstract

**Background:**

The immune microenvironment plays a critical role in maintaining skin homeostasis, which is closely related to the dysfunction in photoaged skin such as autoimmunity and tumorigenesis. Several recent studies have demonstrated the efficacy of 5-aminolevulinic acid photodynamic therapy (ALA-PDT) in alleviating photoaging and skin cancer. However, the underlying immune mechanisms and the immune microenvironment change by ALA-PDT remain largely unknown.

**Methods:**

To illustrate the effects of ALA-PDT on immune microenvironment in photoaged skin, single cell RNA sequencing (scRNA-seq) analysis of photoaged skin on the extensor side of the human forearm before and after ALA-PDT was performed. R-packages of *Seurat, clusterProfiler, Monocle, CellChat* were used for cell clustering, differentially expressed genes analysis, functional annotation, pseudotime analysis and cell-cell communication analysis. The gene sets related to specific functions were extracted from the MSigDB database, which were used to score the functions of immune cells in different states. We also compared our result with published scRNA-seq data of photoaged skin of the eyelids.

**Results:**

The increase score of cellular senescence, hypoxia and reactive oxygen species pathway in immune cells and the decrease of immune receptor activity function and proportion of naive T cells were found in skin photoaging. Moreover, the function of T cell ribosomal synthesis was also impaired or down regulated and function of G2M checkpoint was up regulated. However, ALA-PDT showed promising results in reversing these effects, as it improved the above functions of T cells. The ratio of M1/M2 and percentage of Langerhans cells also decreased with photoaging and increased after ALA-PDT. Additionally, ALA-PDT restored the antigen presentation and migration function of dendritic cells and enhanced cell-cell communication among immune cells. These effects were observed to last for 6 months.

**Conclusion:**

ALA-PDT has potential to rejuvenate immune cells, partially reversed immunosenescence and improved the immunosuppressive state, ultimately remodelling the immune microenvironment in photoaged skin. These results provide an important immunological basis for further exploring strategies to reverse skin photoaging, chronological aging and potentially systemic aging.

## 1 Introduction

Skin, as the body’s largest and outermost organ, is subjected to both intrinsic and extrinsic factors that contribute to aging. Intrinsic factors such as genetic factors, the accumulation of senescent cells, and senescence-associated secretory phenotype (SASP) can contribute to aging, while environmental factors such as ultraviolet radiation (UVR) can cause extrinsic aging, commonly known as photoaging (1). Besides its effect on appearance, photoaging caused by UVR can alter skin microenvironment, especially immune microenvironment, thereby promoting occurrence and development of various skin diseases including skin cancers (2). Photoaging as well as skin chronological aging can also spread the aging phenotype to other tissues or organs, inducing systemic aging (3). Therefore, improving skin photoaging is key to preventing tumorigenesis, promoting elderly skin health, and delaying skin and even systemic aging.

At present, treatment of skin photoaging can be categorized as medical treatment, chemical and physical peeling treatment, photoacoustic and electrical therapy and injection (4). Photodynamic therapy (PDT) is a newer treatment that uses a photosensitizer excited by irradiation of a specific wavelength of laser to treat various conditions, such as photoaging, cancer and acne (5). PDT is advantageous due to its non-invasive or minimally invasive nature, low toxicity, good local selectivity, reproducibility, better safety profile and low resistance. Clinical research by Wang et al. has found that the rejuvenation effects of ALA-PDT including improvement of photoaging appearance can last up to 2 years (6, 7).

Adverse reactions such as mild local skin erythema and swelling sometimes occur after PDT, implying that PDT can change immunological response. Although immune cells in the traditional sense make up only a small part of skin tissue (about 5% according to our single-cell data), they perform a huge and promising function. During photoaging, skin immune microenvironment changes significantly. UVR exposure induces and aggravates cellular senescence, especially in keratinocytes (KC) and fibroblasts (FB) which can promote cytokines and SASP release (8, 9). These changes affect cell function and intercellular communication, disrupting tissue homeostasis, resulting in chronic inflammation and immunosuppressive microenvironments (10). In addition, during aging, the body’s ability to respond effectively to new stimuli is impaired, and the function of the immune system is reduced, especially adaptive immunity, which is called immunosenescence. The hallmark of immunosenescence is the decrease in proportion of naive T cells caused by thymic involution. During this process, there is a decrease in the antigen presentation function of dendritic cells (DC) and macrophages (Mø), as well as a reduction in the number and phagocytosis functions of Mø; comparatively, the number and function of myeloid-derived suppressor cells (MDSCs) increase (11). Wang et al. have found that ALA-PDT can trigger macrophage polarization to the M1 pro-inflammatory phenotype and induce maturation of DCs when treating skin cancer and acne (12–14). Recent findings by our group showed that ALA-PDT increases the density of skin lymphatic and blood vessels and the rate of lymphatic clearance during the treatment of skin intrinsic aging (15). Moreover, our research demonstrated that during photoaging treatment, PDT can reshape dermal collagen by altering the functions of KC and FB (16, 17). However, the immunological mechanisms behind ALA-PDT for photoaging remain largely unknown and therefore requires further elucidation.

To investigate the impact of ALA-PDT on the skin immune microenvironment during photoaging treatment, scRNA-seq analysis before and after treatment were performed. Immune cells in the traditional sense were extracted for further analysis in this study, and the interaction between immune cells and KC, FB, etc. is the direction of our further research. Our results demonstrated that in addition to changing the proportion of immune cell subtypes, ALA-PDT can also improve the cellular senescence degree, hypoxia state, immune receptor activity, reactive oxygen species (ROS) pathway and cell-cell communication of immune cells, and ribosomal synthesis function of T cells (TC) and enhance the antigen processing and presentation function of DC. Moreover, we also found that the proportion of naive T cells and M1/M2 in skin decreased during photoaging, which seemed to suggest that immunosenescence can affect the skin, and ALA-PDT increased the above proportion. In conclusion, PDT could rejuvenate immune cells and reshape the immune microenvironment by changing the composition and functions of immune cells, including TC, Mø, DC and Langerhans cells (LC). These results contribute to the development of novel skin photoaging treatment and potentially preventing or delay skin chronological aging and systemic aging.

## 2 Materials and methods

### 2.1 Photodynamic therapy treatment and tissue acquisition

Two volunteers were recruited (2 females, 54 and 56 years old; Fitzpatrick skin type III or IV) who demonstrated photoaging phenotype on extensor side of forearms and the back of hands according to the Glogau scale (18). After informed consent, 20% ALA cream with a thickness of 1 mm was applied to the skin of left forearm extensor and the back of left hand. And after 30 min of incubation, the ALA-applied area was irradiated by a red LED light (630 nm, 144 J/cm^2^, 40 mW/cm^2^; Philips, Netherlands) for 1 h. The identical ALA-PDT was performed every two weeks for a total of 5 times. The skin samples (3 mm × 3 mm) on the extensor skin of two subjects’ left forearm were taken for scRNA-seq before ALA-PDT, 1 week and 6 months after final ALA-PDT. The sampling interval is about 3 months and 6 months, respectively, and the distance between each sampling site is about 5 cm to ensure that it is not affected by the last sampling.

### 2.2 Tissue dissociation and droplet based single-cell RNA sequencing

Fresh skin tissue was collected from the extensor side of the subject’s forearm with a scalpel. Under sterile conditions, the tissue was washed twice with pre-chilled Roswell Park Memorial Institute (RPMI) 1640 plus 0.04% bovine albumin (BSA) medium. The tissue was cut into small pieces of about 0.5 mm^3^ with surgical scissors, and put into a freshly configured enzymatic digest (2 mg/mL collagenase I, 2 mg/mL collagenase IV, 2 mg/mL dispase and 0.125% trypsin-EDTA). It was enzymatically digest in a constant temperature incubator at 37°C for 30-60 min (19), and mixed upside down every 5-10 min. The digested cell suspension was filtered 1-2 times with BD 40 μm cell sieve, centrifuged at 4°C, 300 g for 5 min. After resuspending the pellet with an appropriate amount of medium, an equal volume of red blood cell lysate (MACS, catalog number 130-094-183) was added, mixed well and stood at 4°C for 10 min. Then the cell suspension was centrifuged at 300g for 5 min, and the supernatant was discarded. The pellet was washed 1 time with medium, centrifuged at 300 g for 5 min and the supernatant was discarded. The cell pellet was resuspended with 100 μl of medium, and the cell concentration and viability were calculated by a Luna cell counter.

The freshly prepared single-cell suspension was adjusted to the concentration of 700-1200 cells/μl, and the library were constructed according to the 10× Genomics Chromium Next GEM Single Cell 3' Reagent Kits v3.1 (catalog number: 1000268) operating instructions. The constructed library used the Illumina Nova 6000 PE150 platform to perform the high-throughput sequencing.

### 2.3 scRNA-seq data processing and cell type identification

After sequencing, raw FASTQ files were then processed by 10×CellRanger using the Human reference genome NCBI build 38 (GRCh38) to generate gene expression matrix for each sample. Then further analyses were performed by the *Seurat* r-package (V4.1.1) (20). A total of 73,763 cells were analyzed. Cells with more than 50,000 total unique molecular identifier count, less than 500 genes, more than 6,000 genes, more than 25% mitochondrial genes, more than 1% hemoglobin genes or more than 3% ribosome genes were filtered out as low-quality cells in the downstream analysis. After quality control, 59812 cells remained for further analysis. NormalizeData function and FindVariableFeatures functions were used to normalize the expression data and to identify variable genes with default parameters before data integration, respectively. Then, FindIntegrationAnchors and IntegrateData functions were used to integrate data for eliminating potential batch effects. After integration, the ScaleData function was used to scale and center the expression counts. Principal components analysis (PCA) was performed on the variable genes, and the first 25 PCs were used to define cell clusters by FindNeighbors and FindClusters functions with the resolution set to 0.2. Uniform manifold approximation and projection (UMAP) dimensional reduction was performed using the RunUMAP function for visualization. The cluster marker genes were identified by the FindAllMarkers function with default parameters. A total of 14 cell clusters were annotated, from which we selected 2543 immune cells for further analysis. The cell types were annotated by overlapping the cluster markers with the canonical cell type signature genes, including lymphocyte (*CD3D, CXCR4*), dendritic cell (*HLA-DRA, CD74*) and mast cell (*TPSB2, TPSAB1*). These three groups of cells were re-clustered by the FindClusters function with the resolution set to 0.2 and 7 immune cell clusters were defined by the same method as mentioned above.

### 2.4 DEGs identification and function annotation

The FindMarkers function in the *Seurat* r-package was used to detect differentially expressed genes (DEGs) of each cell type before and after ALA-PDT treatment. genes with | avg_logFC | > 0.25 and p_val_adj < 0.05 were considered as DEGs. To annotate the functions of DEGs, Gene Ontology (GO) analysis, Kyoto Encyclopedia of Genes and Genomes (KEGG) and gene set enrichment analyses (GSEA) were performed by the *clusterProfiler* r-package. The *Pheatmap* r-package (V1.0.12) was used to visualize the average gene expression of genes among different cell groups and then the *ggstatsplot* r-package (V0.9.5) was used to test the significant difference of gene expression by the chi-square test and together with visualization. The Venn diagram of DEGs from different groups were generated by the jvenn tool (http://bioinfo.genotoul.fr/jvenn) (21).

### 2.5 Pseudotime analysis, gene set score analysis and cell-cell communication analysis

The *Monocle* r-package (V2.24.1) was used to reconstruct CD8+ and CD4+ T cells developmental trajectory. The *irGSEA* r-package (V1.1.3) was used to score and visualize the inflammation response function of different immune cell groups. The *UCell* (V2.3.0) and *AUCell* (V1.18.1) r-package were used to score and visualize other module scores from the MSigDB database (https://www.gsea-msigdb.org/gsea/msigdb/). The *CellChat* r-package (V1.6.0) was used to analysis of cell-cell communication before and after ALA-PDT.

### 2.6 Photoaging and chronological aging scRNA-seq data analysis

Photoaging scRNA-seq data from the human upper eyelid skin (2 mm × 15 mm) from donors that are divided 3 groups of young (18-28 years old), middle-aged (35-48 years old), and old (70-76 years old) was also analyzed (https://bigd.big.ac.cn/aging/landscape?project=Human_Skin) (19). And chronological aging scRNA-seq data from the human inguinoiliac skin (4-mm punch) from young (25-27 years old) and old (53-70 years old) donors recently published (GSE130973) (22). Immune cells were selected for similar analysis as above.

## 3 Results

### 3.1 Changes in the proportion and functions of immune cells in photoaging and following ALA-PDT treatment

**Figure 1:**
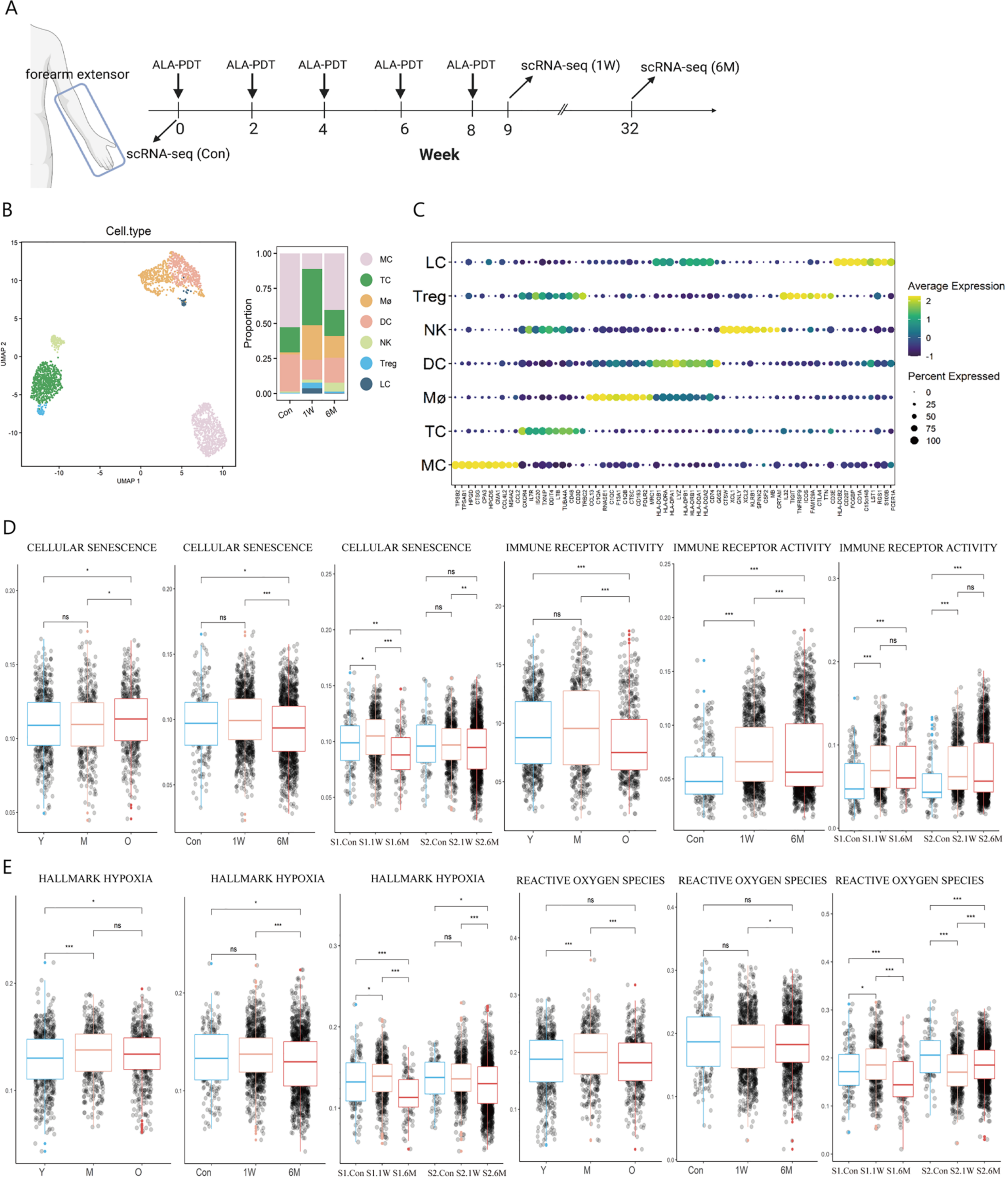
Immune cell type identification in ALA-PDT and changes in cell ratio and functions before and after treatment. **(A)** Schematic diagram of the ALA-PDT process. The extensor forearms and the back of hands were treated every two weeks, with scRNA-seq performed before treatment (Con) and Iat 1 week (1W) and 6 months (6M) after final treatment. **(B)** UMAP plot visualizing immune cells in ALA-PDT (left). Bar chart of changes in the proportion of immune cell subsets in ALA-PDT (right). **(C)** Circle plot of markers of different cell subpopulations. **(D, E)** Boxplots of AUCell scores of GO or HALLMARK pathway of immune cells (* P.adj<0.05, **P.adj<0.01, *** P.adj<0.001, by Wilcox test). (Con, 1W and 6M represented the photoaged skin before PDT, 1 week and 6 months after PDT, respectively. Y, M and O represented the photoaged skin from the young, middle-aged and old, respectively.).

Two subjects with photoaging phenotype on extensor forearms and the back of hands received ALA-PDT every two weeks for a total of 5 sessions. After the treatment, patients felt that their skin became smoother, hydrated and wrinkles were reduced (**Supplementary Figure 1**). Since the rejuvenating effect of ALA-PDT can last for 2 years (6, 7), we continued to observe the long-term effects of PDT for 6 months. As a result, skin biopsies were taken before treatment (Con) as well as 1 week (1W) and 6 months (6M) post-therapy for scRNA-seq analysis (**Figure 1A**). After quality control (**Supplementary Figure 2A**), 59812 cells remained and 2543 immune cells were extracted for further analysis including MC (*TPSB2, TPSAB1*), T cells (TC) (*CD3D, CXCR4*), Mø (*CD163, C1QA*), DC (*HLA-DRA, HLA-DPA1*), natural killer cells (NK) (*CTSW, GNLY*), regulatory T cells (Treg) (*CD3D, FOXP3*), and LC (*CD1A, CD207*) (**Figures 1B, C**). Due to the small number of Treg cells, they were later combined with TC in the subsequent analysis. Functional annotation analysis demonstrated that all immune cells participated in the inflammatory response pathway (**Supplementary Figure 2B**).

Published scRNA-seq analysis by Liu et al. and Lyko et al. showed that the score of cellular senescence of immune cells increased but the score of immune receptor activity decreased significantly during photoaging, while ALA-PDT can improve cellular senescence at 6M and restored immune receptor activity function at 1W and lasted at least 6 months, which suggest that ALA-PDT can rejuvenate immune cells for at least 6 months (**Figure 1D**). Since PDT can produce ROS, we further assessed the degree of hypoxia and ROS pathway in immune cells at different stages. It was found that the hypoxia score in photoaged immune cells increased since middle age, which was relieved at 6M after ALA-PDT (**Figure 1E**). Interestingly, the ROS pathway peaked in the photoaging middle-aged group (35-48 years old), but decreased to the young level in old group. However, volunteers of this study (54 and 56 years old) approached the middle-aged group, and ALA-PDT reduced the transcription level of genes associated with the ROS pathway at 6M, suggesting the long-term protective effect of ALA-PDT (**Figure 1E**).

In photoaging, the proportion of LC and Mø decreased, while the proportion of DC was the largest in the middle age, but decreased in the old age (**Supplementary Figure 2C**). The proportion of T cells (including Treg), Mø and LC increased at 1W compared with Con; while the trend excepted TC persisted for 6 months (**Figure 1B** right, **Supplementary Figure 2D**).

### 3.2 T sub-clusters in ALA-PDT on photoaged skin and photoaging at single cell resolution

**Figure 2:**
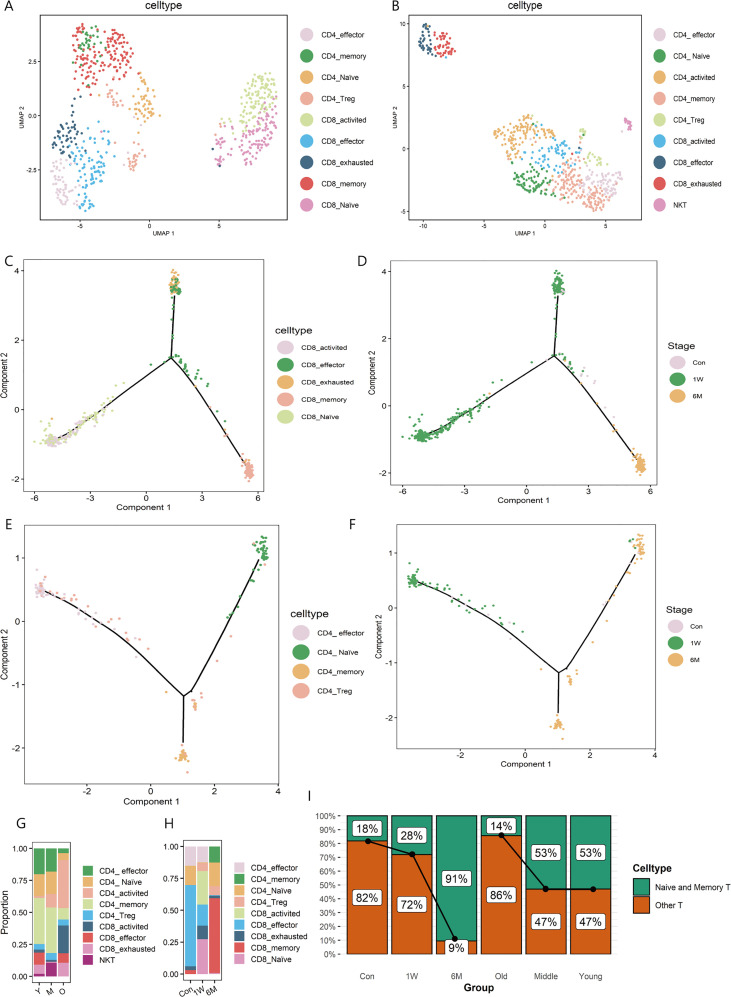
T sub-clusters in ALA-PDT and photoaging. **(A, B)** UMAP plots visualizing the T sub-clusters in ALA-PDT and photoaging, respectively. **(C, D)** Pseudotime analysis of CD8+T cells in ALA-PDT marked by cell type and stage, respectively. **(E, F)** Pseudotime analysis of CD4+T cells in ALA-PDT marked by cell type and stage, respectively. **(G, H)** Bar charts of changes in the proportion of T cell subsets in photoaging and ALA-PDT, respectively. **(I)** Bar chart of changes in the proportion of naive and memory T cells by ALA-PDT and photoaging.

The T cells subpopulations both in ALA-PDT on photoaged skin and photoaging were further analyzed. In ALA-PDT, there were CD4_effector (*GZMA*), CD4_memory (*CD69, CD44*), CD4_Naive (*CCR7*), CD4_Treg (*TIGIT, FOXP3*), CD8_activited (*CD28, CD69*), CD8_effector (*GZMA*), CD8_exhausted (*CTLA4*), CD8_memory (*CD44, CD69*) and CD8_Naive (*CCR7, RPS13*) (**Figure 2A**, **Supplementary Figure 3A**). In photoaging, there were CD4_effector (*CD4*, *GZMA*), CD4_memory (*CD4*, *ITGAE*), CD4_Naive (*CD4*, *TCF7*), CD4_Treg (*PDCD1, FOXP3*), CD4_activited (*CD4*, *CD28)*, CD8_activited (*CD8B, CD28*), CD8_effector (*CD8B, GZMA*), CD8_exhausted (*CD8B, HAVCR2, PRF1*) and NKT (*NKG7, CD8B*) (**Figure 2B**, **Supplementary Figure 3B**).

Pseudotime analysis revealed that CD8+ T cells differentiated into two branches from naive and activated states (**Figure 2C** bottom left), one as effector and exhausted T cells (**Figure 2C** top) and the other as memory T cells (**Figure 2C** bottom right).Accordingly, pre-treatment CD8+ T cells were mainly on the upper branches (effector and exhausted T cells) (**Figure 2D**, **Supplementary Figure 3C** top) and CD8+ T cells at 1 week and 6 months post-therapy were mainly distributed in the lower left and lower right branch (the naive and memory T cells, respectively) (**Figure 2D**, **Supplementary Figure 3C** top) when cells were labelled by treatment status. Similarly, CD4+ T cells pseudotime analysis also showed the differentiation from the naive state of two branches: effector and exhausted T cells and memory T cells (**Figure 2E**). Additionally, 6 months after treatment CD4+ T cells were mainly distributed on the branches of naive and memory T cells (**Figure 2F**, **Supplementary Figure 3C** bottom).

Further investigating the changes in the proportion of T cells subsets, we found that ALA-PDT altered skin T cells subpopulation composition, especially naive T cells. In photoaging, the major constituent of T cells changed from memory and naive cells to activated T cells (**Figures 2G, I**). However, before ALA-PDT, effector T cells predominated, but after ALA-PDT, memory T cells and naive T cells predominated (**Figures 2H, I**). In particular, the ratio of CD4+ naive T cells decreased during photoaging, while it decreased first at 1W but increased significantly at 6M (**Figures 2G, H**). Besides, the proportion of CD8+ T cells increased in photoaging compared with young, which was lower at 6M (**Figures 2G, H**). There are studies reported that the proportion of naive T cells and the proportion of CD4+ T/CD8+ T decreased during the process of immunosenescence (11, 23). And these results indicated that immunosenescence can occur in photoaged skin and can be reversed by ALA-PDT.

### 3.3 ALA-PDT restored T cell ribosome biogenesis function

**Figure 3:**
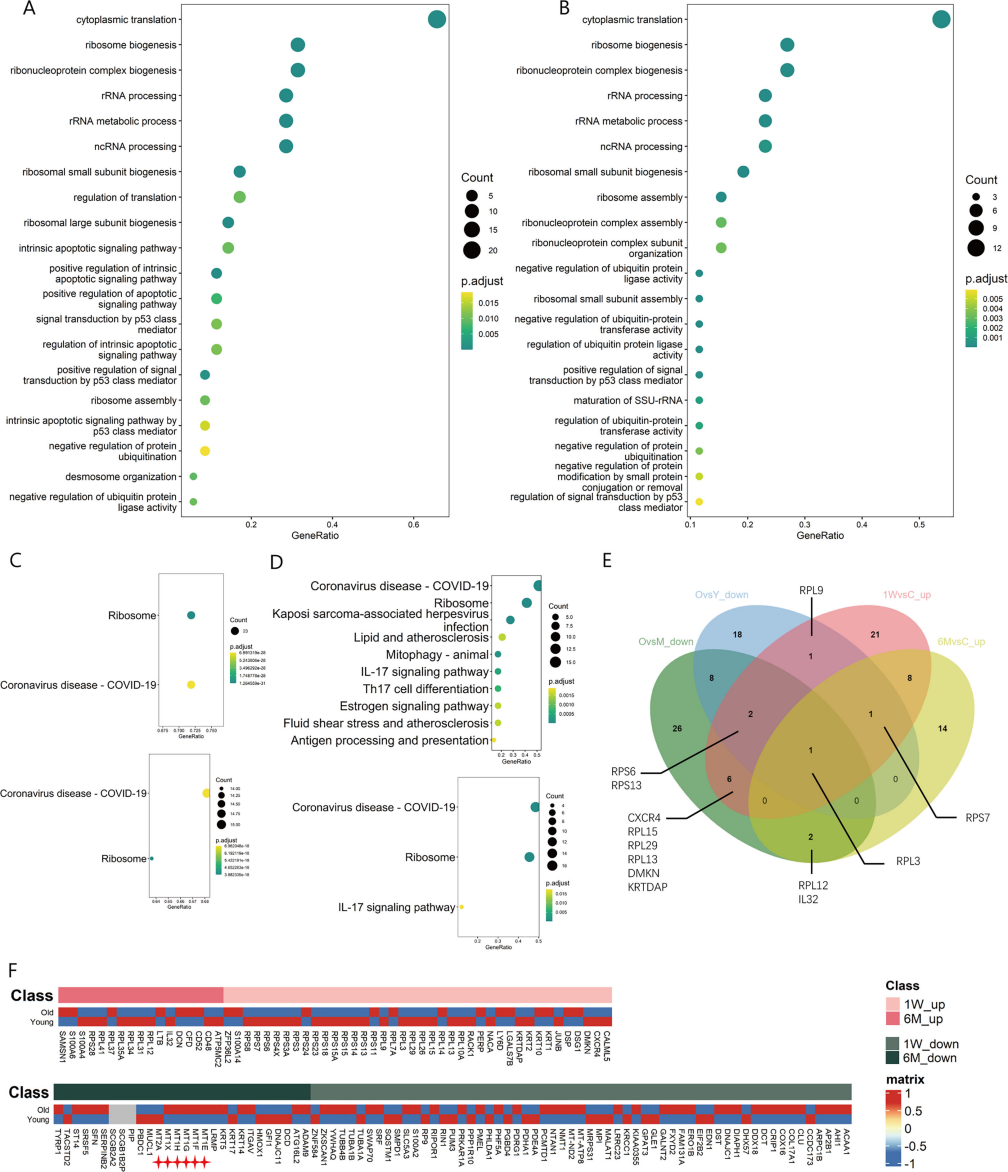
Changes of ribosome biogenesis function of T cells in ALA-PDT and photoaging. **(A, B)** GO function enrichment analysis of DEGs upregulated of T cells in 1W/Con and 6M/Con in ALA-PDT, respectively. **(C)** KEGG function enrichment analysis of DEGs upregulated of T cells in 1W/Con (top) and 6M/Con (bottom) in ALA-PDT. **(D)** KEGG function enrichment analysis of DEGs downregulated of T cells in Old/Young (top) and Old/Middle (bottom) in photoaging. **(E)** Venn plot of DEGs upregulated of T cells in ALA-PDT and DEGs downregulated in photoaging. **(F)** Heatmaps of the expression of up-regulated (top) and down-regulated (bottom) DEGs in T cells by ALA-PDT in photoaged T cells.

Function enrichment analysis of T cell DEGs, regardless of 1W or 6M post-PDT, upregulated gene functions were mainly enriched in ribosome synthesis related terms like “ribosome biogenesis”, “rRNA processing” and “ribosome assembly” in GO analysis and “Ribosome” in KEGG analysis (**Figures 3A–C**). Nevertheless, in photoaged skin, down-regulated gene functions of T cells were also enriched in the ribosome synthesis related term when compared with young or middle-aged skin (**Figure 3D**). DEGs upregulated after ALA-PDT overlaps with the genes downregulated during photoaging, which include ribosomal proteins: *RPL3*, *RPL12*, *RPL13*, *RPL15*, *RPL29, RPS6*, *RPS7* and *RPS13* (**Figure 3E**).

To explore the relationship of between T cells in ALA-PDT and photoaging, heatmaps were generated based on the expression of DEGs of T cells in ALA-PDT in both groups. ALA-PDT up-regulated DEGs were mainly expressed in the young group, especially the genes involved in ribosome biogenesis, while the ALA-PDT down-regulated genes were mainly expressed in old group from photoaging study (**Figure 3F**). These results indicate that ALA-PDT can reverse the impaired ribosomal biogenesis function of T cells during photoaging and may rejuvenate T cells. Interestingly, the ribosomal biogenesis function of T cells was also reduced during intrinsic aging (**Supplementary Figures 3D, E**), suggesting a potential therapeutic role of ALA-PDT in chronological aging.

### 3.4 ALA-PDT rejuvenated T cell functions

**Figure 4:**
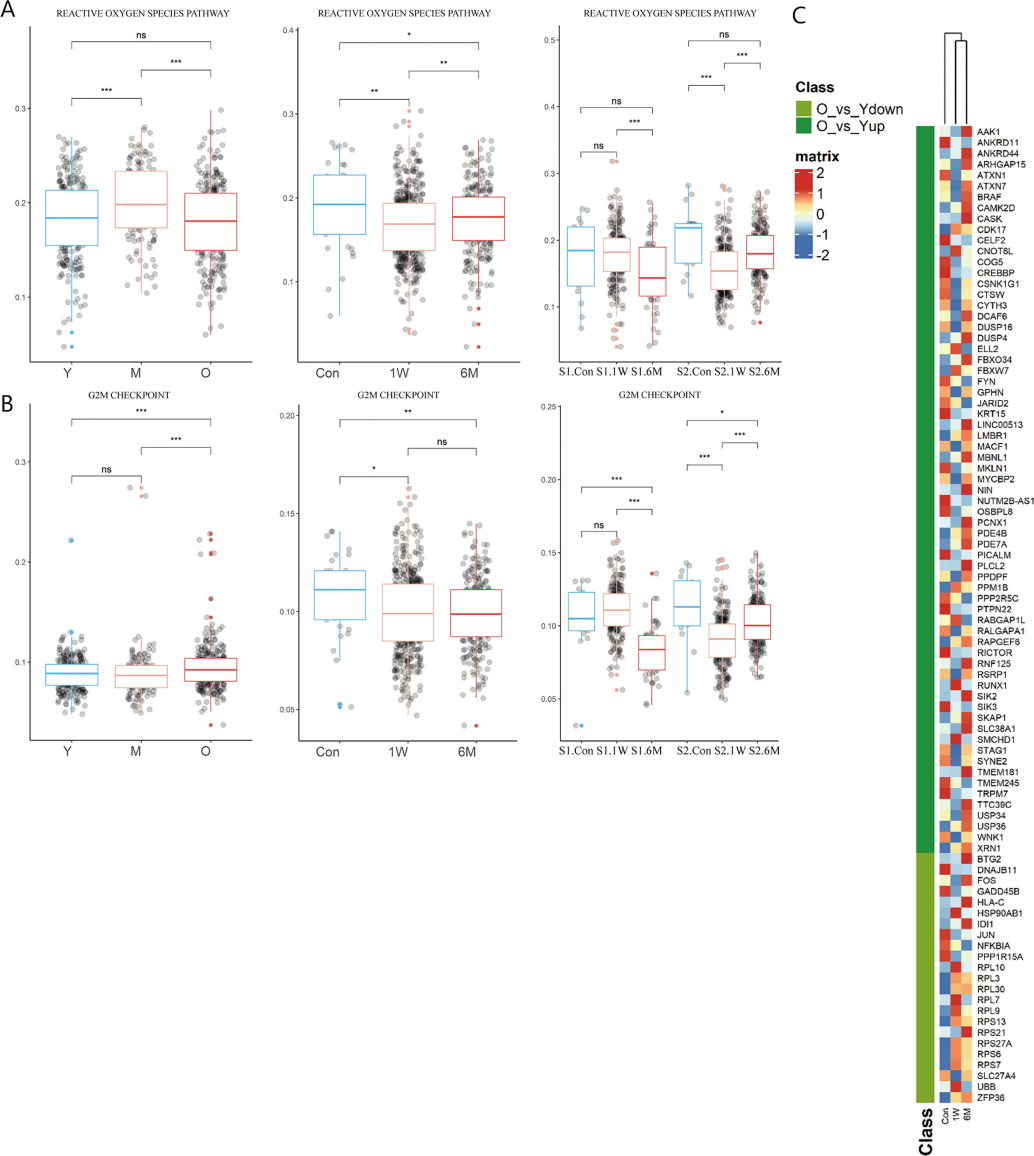
Changes of T cells function in ALA-PDT and photoaging. **(A, B)** Boxplots of AUCell scores of HALLMARK pathways of T cells in ALA-PDT and photoaging (* P.adj<0.05, ** P.adj<0.01, *** P.adj<0.001, by Wilcox test). **(C)** Heatmap of the expression of DEGs of T cells during photoaging in T cells of ALA-PDT.

During photoaging, the score of ROS pathway increased in the middle-aged group, returning to youthful levels in old age. Interestingly, although ALA-PDT is capable of producing ROS, the T cell ROS pathway score decreased at 6M, suggesting the long-term potential advantage of ALA-PDT (**Figure 4A**). Additionally, function of G2M checkpoints of TC was significantly increased in the photoaging elderly group. However, ALA-PDT reversed it and maintained for at least 6 months (**Figure 4B**).

Previous studies have found that metallothioneins (MT), zinc-binding proteins, are overexpressed in the chronic inflammation of aging, which leads to increased zinc conjugation, reducing the available free zinc, thus impairing immunity (24–26). Our result confirmed the upregulation in the expression of *MT-1E, MT-1F, MT-1G, MT-1H* and *MT-1X* in T cells during photoaging. Interestingly, expression of *MTs* was down-regulated after ALA-PDT, which may contribute to the immune function alternation (**Figure 3F** star). And *ZFP36L2* (zinc-finger protein involved in the CD4+ T cell response) was up-regulated after ALA-PDT (**Figure 3F** top) (27).

To further investigate whether T cells after ALA-PDT demonstrated a youthful state, heatmap was plotted based on the expression of DEGs of T cells in photoaging in cells at different stages of ALA-PDT. The T cells after ALA-PDT (including 1W and 6M) were clustered into same class based on cluster analysis (**Figure 4C** top), and DEGs initially downregulated during photoaging were highly expressed after ALA-PDT. Genes with high expression after ALA-PDT accounted for 73.91% of the DEGs upregulated in young T cells in photoaged scRNA-seq data (**Figure 4C**). Therefore, ALA-PDT may lead T cells acquiring a youthful phenotype through ribosome synthesis, ROS pathway, G2M checkpoint and activation.

### 3.5 ALA-PDT altered the functions of Mø, LC and DC

**Figure 5:**
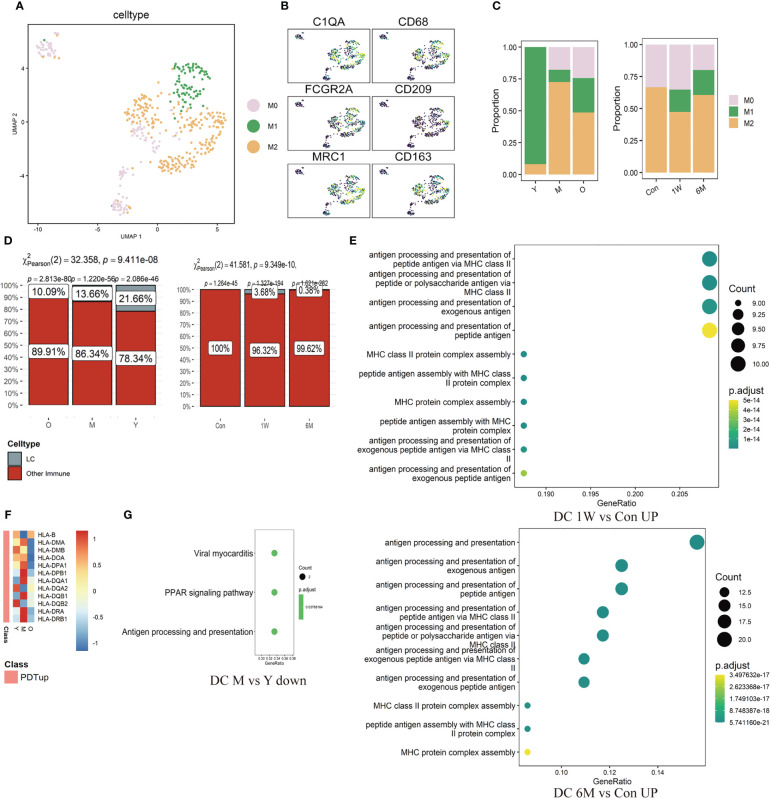
Changes of ratio and function of Mø, LC and DC in ALA-PDT and photoaging. **(A)** UMAP plots visualizing the Mø sub-clusters in ALA-PDT. **(B)** UMAP plots of markers of different Mø subpopulations in ALA-PDT. **(C)** Bar charts of changes in the proportion of Mø subsets in ALA-PDT and photoaging. **(D)** Bar charts of changes in the proportion of LC in photoaging and ALA-PDT, respectively (Pearson’s chi-squared test). **(E)** GO function enrichment analysis of DEGs upregulated of DC in 1W/Con (top) and 6M/Con (bottom) in ALA-PDT. **(F)** Heatmap of the expression of upregulated DEGs in DC by ALA-PDT in photoaging’s DC cells. **(G)** KEGG function enrichment analysis of DEGs downregulated of DC in M/Y in photoaging.

Mø were further divided into M0 (*C1QA*), M1 (*CD68, CD86, FCGR2A*), and M2 (*CD163, CD209, MRC1, CCL18*) subtypes (**Figures 5A, B**, **Supplementary Figures 3F, G**). Proportion of M1/M2 in the photoaged group decreased significantly compared to the young counterpart. After ALA-PDT, the ratio was reversed and continued until 6 months after final treatment (**Figure 5C**). Analysis of LC showed that the proportion of LC in all immune cells gradually decreased with age; from 21.66% in young to 10.09% in photoaged group, indicating immune dysfunction (**Figure 5D** left). Although LC was not detected before treatment, the percentage of LC increased after ALA-PDT, especially at one week after final treatment (**Figure 5D** right).

The antigen-presenting function of DC decreased in the middle-age group compared to the young group in photoaging according to DEGs enrichment analysis (**Figure 5G**). However, ALA-PDT significantly increased this function of DCs at both 1W and 6M (**Figure 5E**, **Supplementary Figure 3H**). In addition, most of the genes upregulated after ALA-PDT were major histocompatibility complex (MHC), and the expression of these genes in photoaged DCs was significantly lower than that in the young or middle-aged group, which also indicated that the antigen presentation function of DC decreased during photoaging and can be improved by ALA-PDT (**Figure 5F**). The score of DC migration appeared to have a decline trend in photoaging, which was increased by ALA-PDT (**Supplementary Figure 3H**).

### 3.6 Cell-cell communication among immune cells

**Figure 6:**
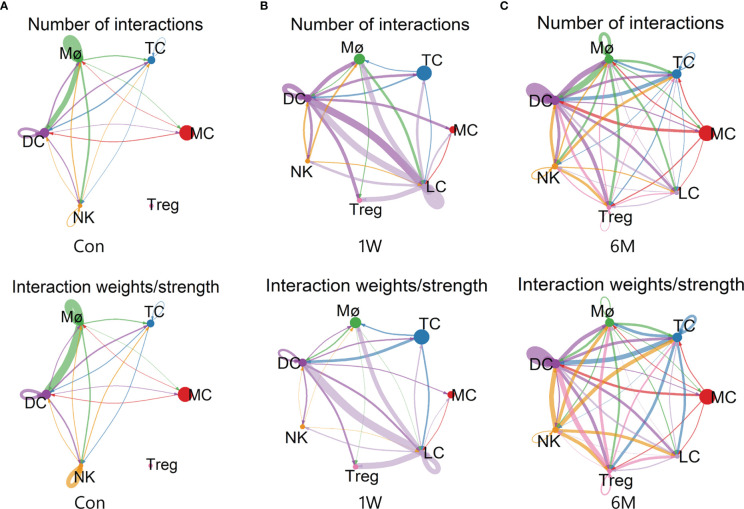
Cell-cell communication between immune cells in ALA-PDT. **(A–C)** Number (top) and strength (bottom) of interactions between different immune cell subtypes before ALA-PDT and at 1 week and 6 months after ALA-PDT, respectively.

Analysis of cellular interactions by *CellChat* r-package showed that the number and strength of communication between immune cells increased significantly after ALA-PDT, and did not decrease at 6 months after treatment. The interactions between Treg and other cells were also correspondingly enhanced. However, the communication strength of Mø and NK decreased after ALA-PDT (**Figure 6**).

## 4 Discussion

Studies have shown that senescent immune cells in aging skin can be detrimental, as they accelerate aging of other organs and promote systemic aging (23). In our study, the score of cellular senescence of immune cells in photoaged skin was significantly higher than young skin. Interestingly, ALA-PDT can effectively rejuvenate immune cells in photoaged skin, which may have beneficial effects on systemic aging. More importantly, these rejuvenating effects by ALA-PDT can sustain for at least 6 months after treatment.

The oxidative stress response produces ROS, which leads to DNA damage and plays a key role in cellular aging (28). Our study found that immune cells in the photoaged elderly group had lower ROS pathway score compared to the middle-aged group, possibly due to reduced responsiveness of immune cells to ROS in photoaged skin. Notably, although ALA-PDT generated ROS to function, the ROS pathway score decreased in both immune cells and T cells at 6M. This may suggest that ALA-PDT first killed senescent cells through ROS which made the cellular senescence score decrease, thereby reducing SASP and other stimuli in the skin microenvironment, and finally reducing the production of ROS and rejuvenating the immune cells.

There is very limited evidence of immunosenescence in the skin. This analysis found that the proportion of naive T and DC functions decreased, cellular senescence score of immune cells and the proportion of M2/M1 increased in photoaged skin. These findings implied that the immune microenvironment of the skin can be influenced by systemic immunosenescence during photoaging. In other words, the immunosuppressive state of photoaged skin may result from a combination of external UVR and systemic immunosenescence.

Previous studies have demonstrated a significant increase in T cells in photoaging (29). However, the change in the ratio of CD4+ T/CD8+ T during skin aging remains controversial. Bulfone-Paus et al. found that the number of MC and CD8+ T cells increased in intrinsic aging of human skin, while CD4+ T cells did not change (30). But Michel et al. found that the proportion of CD4+ T/CD8+ T increased in human aging skin and the proportion of naive T cells decreased (31). The contradiction with the change in the proportion of CD8+ T cell may be due to the difference between photoaging and intrinsic aging. In addition, the proportion of CD4+ T/CD8+ T decreased during the process of immunosenescence (11, 23). Our current study found that the ratio of CD4+ T/CD8+ T decreased during photoaging, which also indicate that systemic immunosenescence affects the photoaged skin, and ALA-PDT increases the percentage of CD4+ T cells.

Studies have shown that both T cells activation and entry into the first cell cycle from G (0) requires ribosome biogenesis (32, 33). Furthermore, many mRNA are strongly repressed in naive T cells, resulting in a large portion of untranslated mRNA. As a result, changes in translation level rather than transcriptional level during T cells activation play a critical role in protein abundance in T cells (34). Naive T cells contain low levels of ribosomal proteins and rRNA, and when T cells are stimulated and activated, the synthesis function of ribosomes can increase to produce the large number of cytokines (35). These findings imply that the increase of T cell number and recovery of immune functions by ALA-PDT may be partially attributed to the restoration of ribosome biogenesis function. Additionally, the increased G2M checkpoint function in photoaging was also reversed by ALA-PDT, which seems to indicate that PDT restores T cells to a youthful state without the need for hyperactive G2M checkpoints.

During aging, both the number and the functions of LC and DC decrease (36–39). In skin aging, LC depletion has been widely recognized (40). In addition, UVR-induced immunosuppression can also be attributed to a decrease in the number and functions of antigen presenting cells (APC) (41). Surprisingly, ALA-PDT restores the number of DC and LC in photoaged skin, recovering the impaired antigen presentation function and migration function of DC in photoaging which can further improve the immune microenvironment.

In conclusion, ALA-PDT has potential to rejuvenate immune cells, partially reverse immunosenescence and improve immunosuppressive state, ultimately reshapes the immune microenvironment. In addition, these therapeutic effects maintained at least 6 months after final treatment. However, the remaining cells (mainly keratinocyte and fibroblasts) also play a role in immune microenvironment which will be investigated in future. Additionally, the exact mechanism how PDT regulates the immune microenvironment remains unknown. There are still unanswered questions including types of immune cells play a central role in PDT-regulated immune microenvironment, and immune cells interactions with other cell types (KC, FB, skin stem cells, etc) in the skin microenvironment.

## Data availability statement

The scRNA-seq data from this study has been uploaded to the Bioproject database, under the accession number PRJNA977409. Furthermore, the published sequencing data for skin photoaging have been uploaded to the Aging Atlas (https://bigd.big.ac.cn/aging/landscape?project=Human_Skin). Additionally, the published sequencing data for intrinsic aging can be found in the Gene Expression Omnibus (GEO) database with the accession number GSE130973.

## Ethics statement

The studies involving human participants were reviewed and approved by The Ethics Committee of the Shanghai skin disease Hospital. The patients/participants provided their written informed consent to participate in this study.

## Author contributions

XW, PW: Ideas, formulation or evolution of overarching research goals and aims. YY: Writing - original draft and data analysis. GY: Implementation of the computer code and supporting algorithms. ZC: Conducting a research and investigation process and performing the experiments. BW, QZ: Writing - review & editing and providing valuable insights and suggestions during discussions. GZ, LZ, LS: Development or design of methodology, creation of models. QC, CC: Data curation and assisted in data collection and analysis. CL, SJ, XS: Supporting for overall research process and sharing resources, equipment, or facilities. All authors contributed to the article and approved the submitted version.

## Supplementary material

Supplementary Figure 1
**(A)** Characteristic locally magnified skin photographs before and after ALA-PDT. **(B)** Photographs of the dorsal and wrist of the hand before and after ALA-PDT.

Supplementary Figure 2
**(A)** Violin diagrams of feature and count of RNA and the ratio of mitochondria, ribosome, and hemoglobin RNA for each sample before (left) and after (right) quality control. **(B)** Ridge plots of HALLMARK-specific pathways in ALA-PDT **(C)** Bar charts of changes in the proportion of immune cell subsets during photoaging. **(D)** Bar charts of changes in the proportion of immune cell subsets for each sample before and after ALA-PDT. (XXQ0, simple1 Con; XXQ2, simple1 1W; XXQ5, simple1 6M; XYZ1, simple2 Con; XYZ2, simple2 1W; XYZ3, simple2 6M)

Supplementary Figure 3
**(A, B)** UMAP plots of markers of different T cells subpopulations in ALA-PDT and photoaging, respectively. **(C)** Pseudotime analysis of CD8+T cells (top) and CD4+T (bottom) cells in ALA-PDT marked by cell type and separated by stage, respectively. **(D, E)** GO and KEGG function enrichment analysis of DEGs down-regulated of T cells in Old/Young in intrinsic aging, respectively. **(F)** UMAP plots visualizing the Mø sub-clusters in photoaging. **(G)** UMAP plots of markers of different Mø subpopulations in photoaging. **(H)** Boxplots of AUCell scores of GO pathways of DC in ALA-PDT and photoaging (* P.adj<0.05, ** P.adj<0.01, *** P.adj<0.001, by Wilcox test).
