# Supplementary figures and images for: Single cell transcriptome profiling reveals cutaneous immune microenvironment remodeling by photodynamic therapy in photoaged skin
Source: Front Immunol. 2023 Jun 19;14:1183709. doi: 10.3389/fimmu.2023.1183709 (PMC10315469; doi:10.3389/fimmu.2023.1183709)

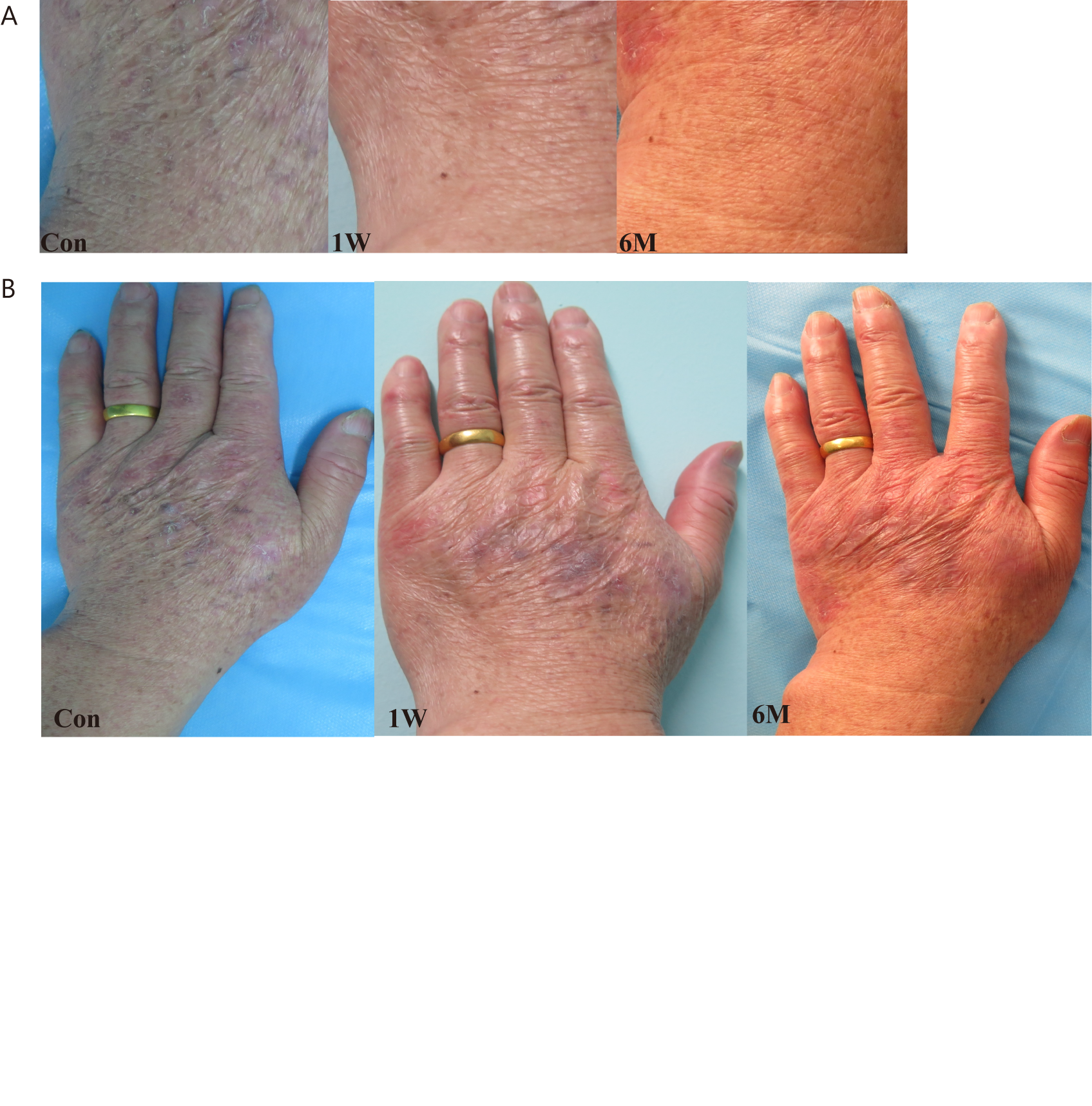

Supplement: Supplementary Figure 1 — (A) Characteristic locally magnified skin photographs before and after ALA-PDT. (B) Photographs of the dorsal and wrist of the hand before and after ALA-PDT. [file Image_1.tif]

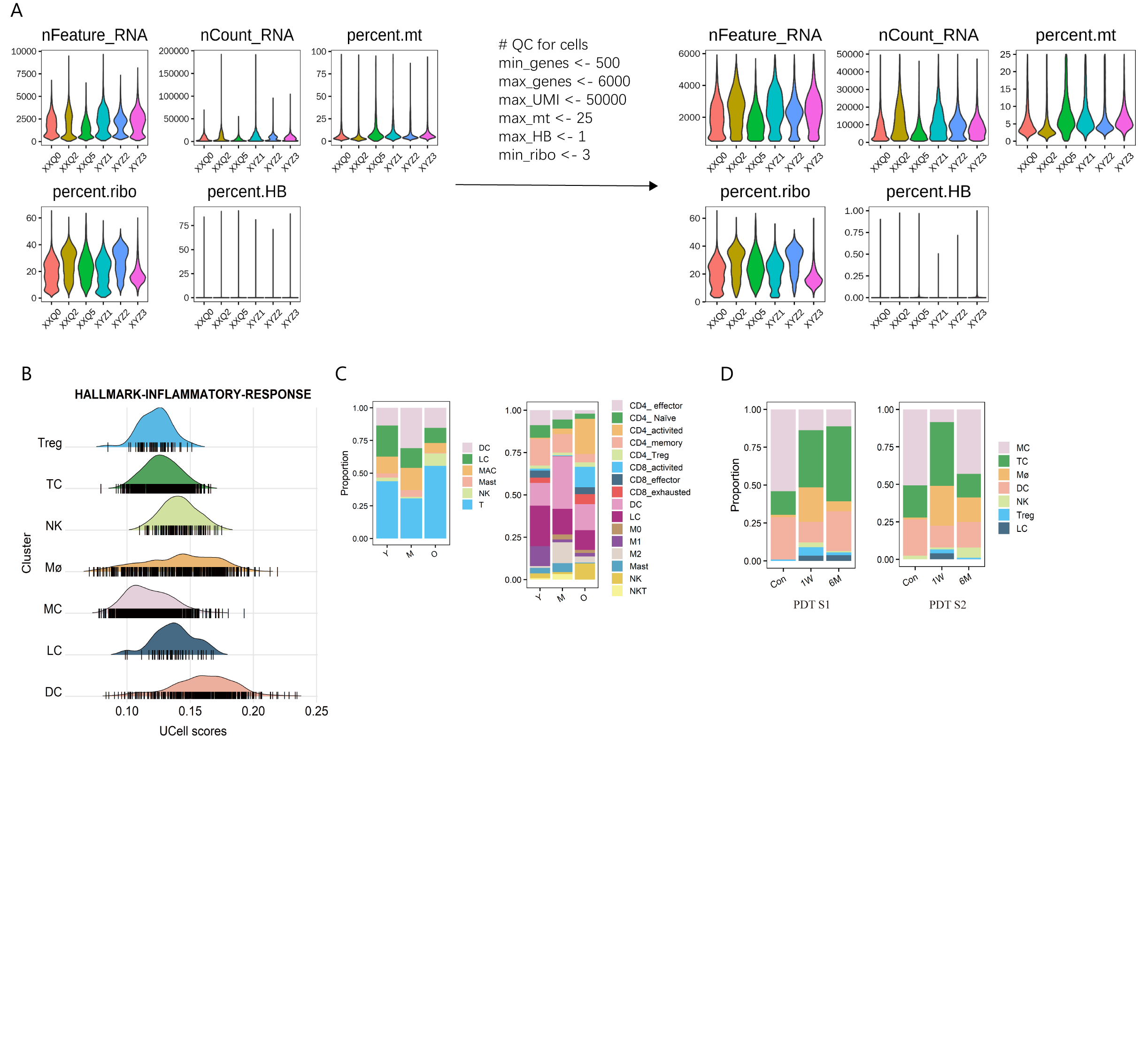

Supplement: Supplementary Figure 2 — (A) Violin diagrams of feature and count of RNA and the ratio of mitochondria, ribosome, and hemoglobin RNA for each sample before (left) and after (right) quality control. (B) Ridge plots of HALLMARK-specific pathways in ALA-PDT (C) Bar charts of changes in the proportion of immune cell subsets during photoaging. (D) Bar charts of changes in the proportion of immune cell subsets for each sample before and after ALA-PDT. (XXQ0, simple1 Con; XXQ2, simple1 1W; XXQ5, simple1 6M; XYZ1, simple2 Con; XYZ2, simple2 1W; XYZ3, simple2 6M) [file Image_2.tif]

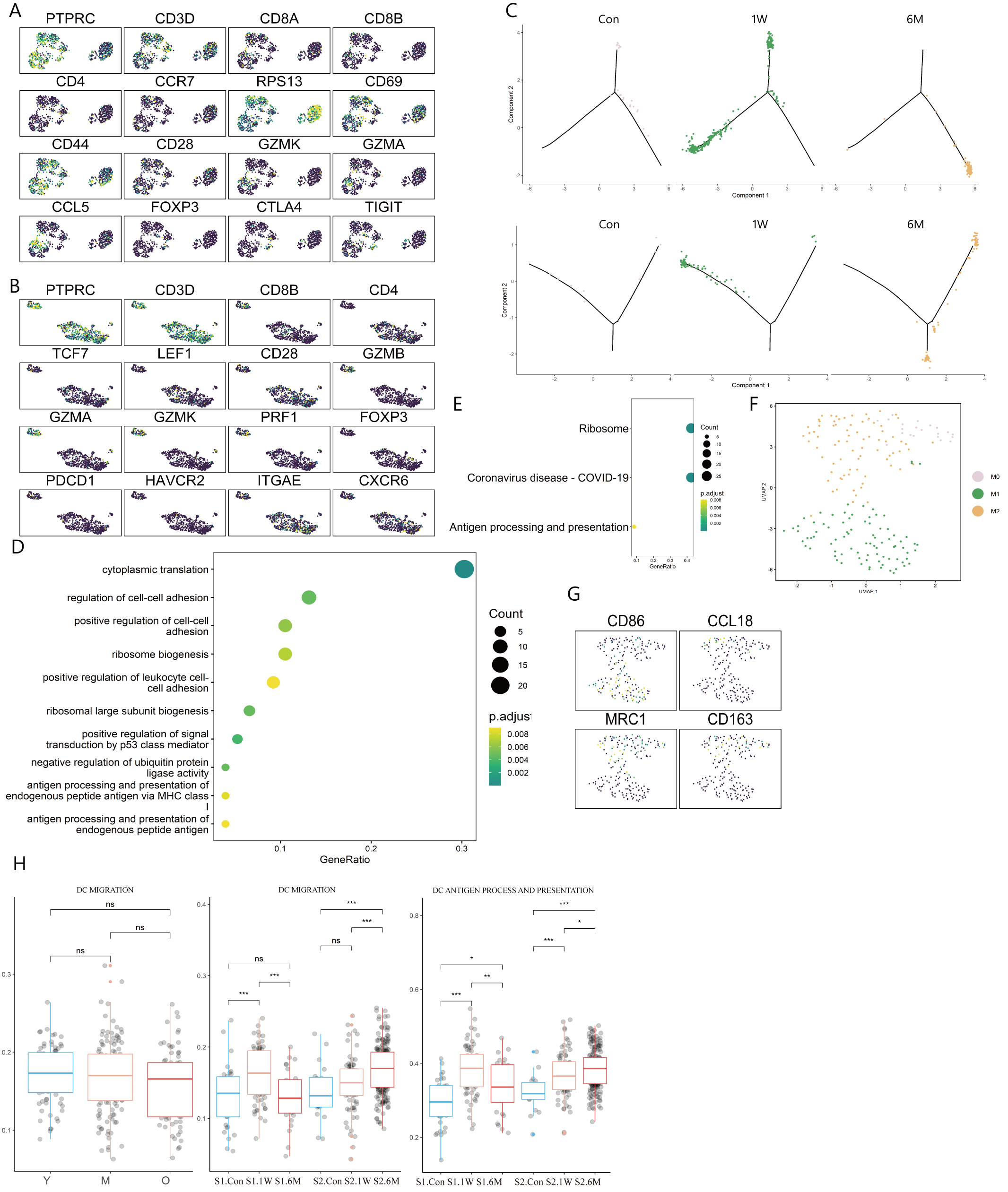

Supplement: Supplementary Figure 3 — (A, B) UMAP plots of markers of different T cells subpopulations in ALA-PDT and photoaging, respectively. (C) Pseudotime analysis of CD8+T cells (top) and CD4+T (bottom) cells in ALA-PDT marked by cell type and separated by stage, respectively. (D, E) GO and KEGG function enrichment analysis of DEGs down-regulated of T cells in Old/Young in intrinsic aging, respectively. (F) UMAP plots visualizing the Mø sub-clusters in photoaging. (G) UMAP plots of markers of different Mø subpopulations in photoaging. (H) Boxplots of AUCell scores of GO pathways of DC in ALA-PDT and photoaging (* P.adj<0.05, ** P.adj<0.01, *** P.adj<0.001, by Wilcox test). [file Image_3.tif]
